# Characterization of radiation-resistance mechanism in *Spirosoma montaniterrae* DY10^*T*^ in terms of transcriptional regulatory system

**DOI:** 10.1038/s41598-023-31509-8

**Published:** 2023-03-23

**Authors:** Changyun Cho, Dohoon Lee, Dabin Jeong, Sun Kim, Myung Kyum Kim, Sathiyaraj Srinivasan

**Affiliations:** 1 Interdisciplinary Program in Bioinformatics, Seoul National University, Seoul, 08826 Republic of Korea; 2 Bioinformatics Institute, Seoul National University, Seoul, 08826 Republic of Korea; 3 BK21 FOUR Intelligence Computing, Seoul National University, Seoul, 08826 Republic of Korea; 4 Department of Computer Science and Engineering, Seoul National University, Seoul, 08826 Republic of Korea; 5 Department of Bio & Environmental Technology, College of Natural Science, Seoul Women’s University, Seoul, 01797 Republic of Korea

**Keywords:** Computational biology and bioinformatics, Computational models, Gene regulatory networks, Machine learning

## Abstract

To respond to the external environmental changes for survival, bacteria regulates expression of a number of genes including transcription factors (TFs). To characterize complex biological phenomena, a biological system-level approach is necessary. Here we utilized six computational biology methods to infer regulatory network and to characterize underlying biologically mechanisms relevant to radiation-resistance. In particular, we inferred gene regulatory network (GRN) and operons of radiation-resistance bacterium *Spirosoma montaniterrae* DY10$^T$ and identified the major regulators for radiation-resistance. Our results showed that DNA repair and reactive oxygen species (ROS) scavenging mechanisms are key processes and Crp/Fnr family transcriptional regulator works as a master regulatory TF in early response to radiation.

## Introduction

Exposure to high levels radiation adversely affects cells, damaging the nucleic acids, proteins, lipids and cellular components of cells, which results in cell death. Ultraviolet (UV) radiation directly damages DNA and causes genetic disruption by inducing pyrimidine dimerization, single-stranded breaks (SSBs) and double-stranded breaks (DSBs)^1–3^. In addition, radiation induces indirect damage through reactive oxygen species (ROS), such as hydroxyl radicals, superoxide anions, and hydrogen peroxide within cells by radiolysis of water. Interestingly, there are radiation-resistant organisms which are able to withstand high levels of radiation exposure. The radiation-resistant organisms are known to protect cells from genome-wide disruption and homeostasis disruption by activating underlying defense systems such as DNA repair and ROS detoxification.

*Deinococcus radiodurans*, is the most well characterized radiation-resistant organism. *Deinococcus radiodurans* shows remarkable radiation-resistance characteristics for high levels of ionizing radiation (IR), X-rays and gamma rays^4^, compared to the yeast *Saccharomyces cerevisiae*, the bacterium *Escherichia coli*, and the human cells. When *Deinococcus radiodurans* radiated, cell recovers the radiation induced damage through efficient regulation of DNA repair systems, including extended synthesis-dependent strand annealing (ESDSA) process and homologous recombination by the RecF pathway^5^. This organism deals with radiation induced ROS through enzymatic systems and non-enzymatic systems, including the following molecules: superoxide dismutase, catalase, peroxidase, pyrroloquinoline-quinone, deinoxanthin, and bacillithiol^6^. In particular, the unusual high intracellular Mn/Fe ratio, has been correlated with radiation-resistance through the low-molecular-weight metabolite (LMWM) systems that forms ROS-scavenging Mn$^{2+}$-metabolite complexes^7^. In addition, it is reported that organisms in three domains of life (Archaea, Bacteria, and Eukaryota) such as *Rubrobacter radiotolerans*, *Kineococcus radiotolerans*, *Halobacterium salinarum*, *Thermococcus gammatolerans* and *Pyrococcus furiosus* have radiation-resistance through different mechanisms^8–11^.

Organism-level biological properties are driven by interactions among genes rather than the effect of individual genes. Genes rarely work individually and generally cooperate to perform specific biochemical functions. Also worth noting is transcription of genes that is mainly regulated by transcription factors (TFs) in a cell-specific manner^12^. Thus, we view that radiation-resistance property is the result of association of multiple intracellular elements including TFs and target genes. Existing studies on radiation-resistance in various strains, such as *Deinococcus radiodurans*, also identified that radiation-resistance is driven through a complex pathways. However, in order to understand the complex underlying mechanism of radiation-resistance both at the transcriptome-level and at the system-level, powerful network-based approaches are needed.

Innovative advances in bioinformatics have accelerated biological knowledge discovery and allowed mathematical modeling of biological systems with transcriptional repressors/activators and post-transcriptional RNA regulators. Ahn et al., applied Gene Regulatory Network (GRN) and successfully identified regulatory modules that respond to cold and heat stress in Arabipodsis^13^. GRN is a collection of genetic molecules that represent systematic understanding of the molecular interactions underlying biological processes includes how gene expressions are regulated and carry out cellular functions^14–17^. GRN based network analysis aids in the identification of regulatory mechanisms to understand complex biological phenomena^12^. For decades, a number of computational methods have attempted to develop GRN inference algorithms^18–21^. One of these methods relies on TF binding motifs or genomic accessibility^22^.

**Figure 1:**
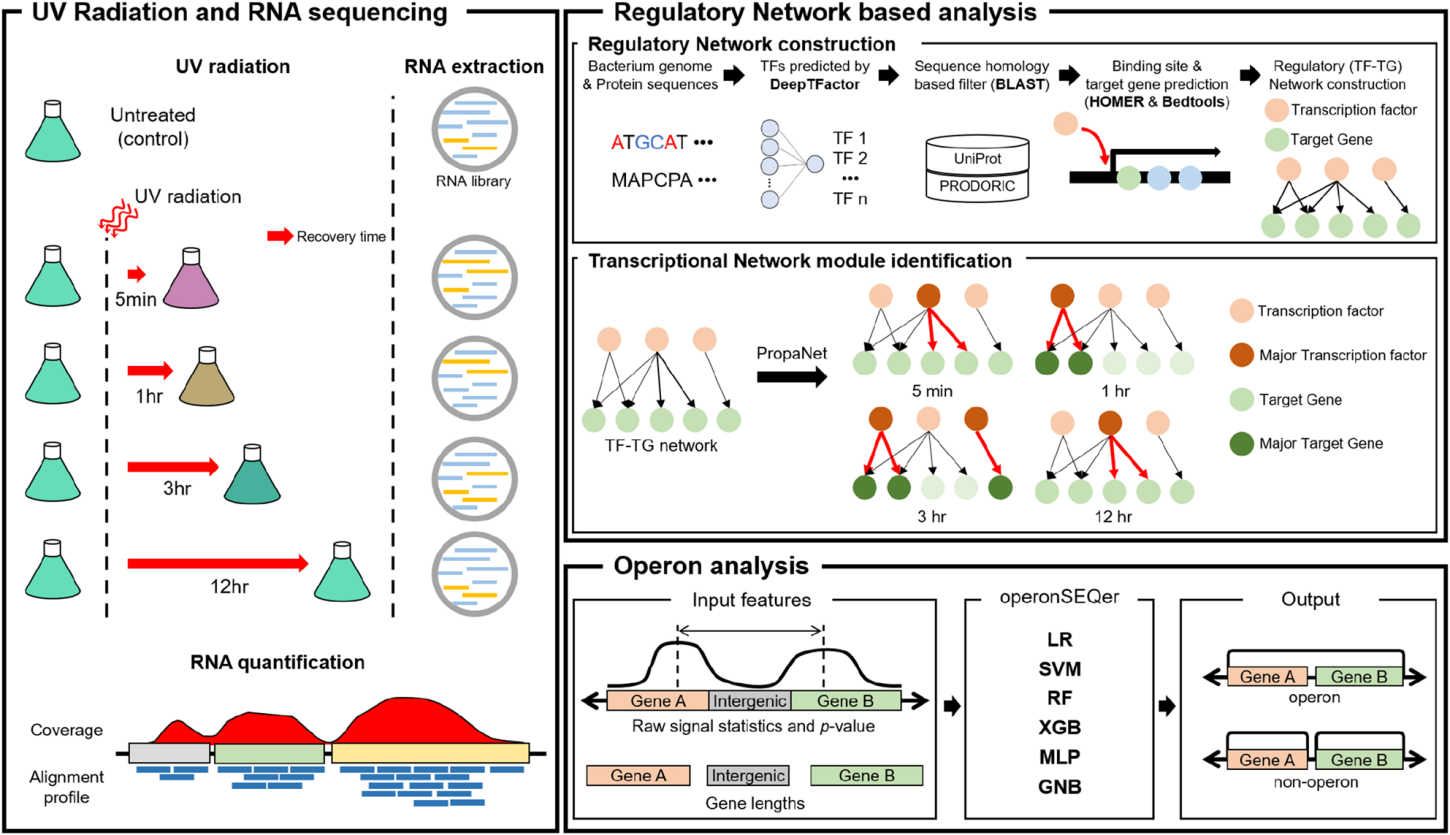
Schematic overview of our approach. In order to investigate radiation-resistance mechanism, RNA from UVC treated and untreated samples was extracted and quantified. In order to understand radiation-resistance mechanism in system-level, regulatory network based analysis and operon analysis were conducted. In the regulatory network based analysis, GRN of *Spirosoma montaniterrae* DY10$^{T}$ was constructed using four computational methods (DeepTFactor, BLAST, HOMER, and Bedtools) and Key regulatory relationships over time on the GRN identified using PropaNet. In the operon analysis, operons in *Spirosoma montaniterrae* DY10$^{T}$ were detected using operonSEQer.

In our previous work, we isolated a remarkable radiation-resistant bacterium *Spirosoma montaniterrae* DY10$^{T}$ from the soil of south Korea and identified their taxon and radiation-resistance property^23^. In addition, several radiation-resistant species have been reported in the *Spirosoma* genus^24,25^. Besides this, the specific mechanism of radiation-resistance of *Spirosoma* genus has not been investigated. Understanding the complex underlying mechanisms of *Spirosoma* genus involves two major issues. First, for understanding complex underlying mechanism, gene-level approach could not bridge the gap between transcriptome-level perturbations and organism-level phenomenon. Second, to apply a system-level approach, knowledge between genes and regulators is limited, and is only available for a few well-known model strains. In this study, we generated time-series transcriptome data of radiation-resistant strain DY10$^T$ under UVC radiation. Subsequently, we show that gene regulatory network for uncharacterized strain can be inferred using six in silico methods without labor-intensive and time-consuming in vitro and in vivo experiments (Fig. 1). Dataflow of utilized computational tools were summarized in Supplementary Table S1. In summary, our biological system-level approach revealed that the radiation-resistance phenotype of strain DY10$^{T}$ stems from DNA repair and ROS scavenging mechanisms, and we identified Crp/Fnr family transcriptional regulator as a master regulatory TF in early response to UVC radiation.

## Results

### Biological implications of *Spirosoma montaniterrae* DY10$^T$ in response to UVC radiation

**Figure 2:**
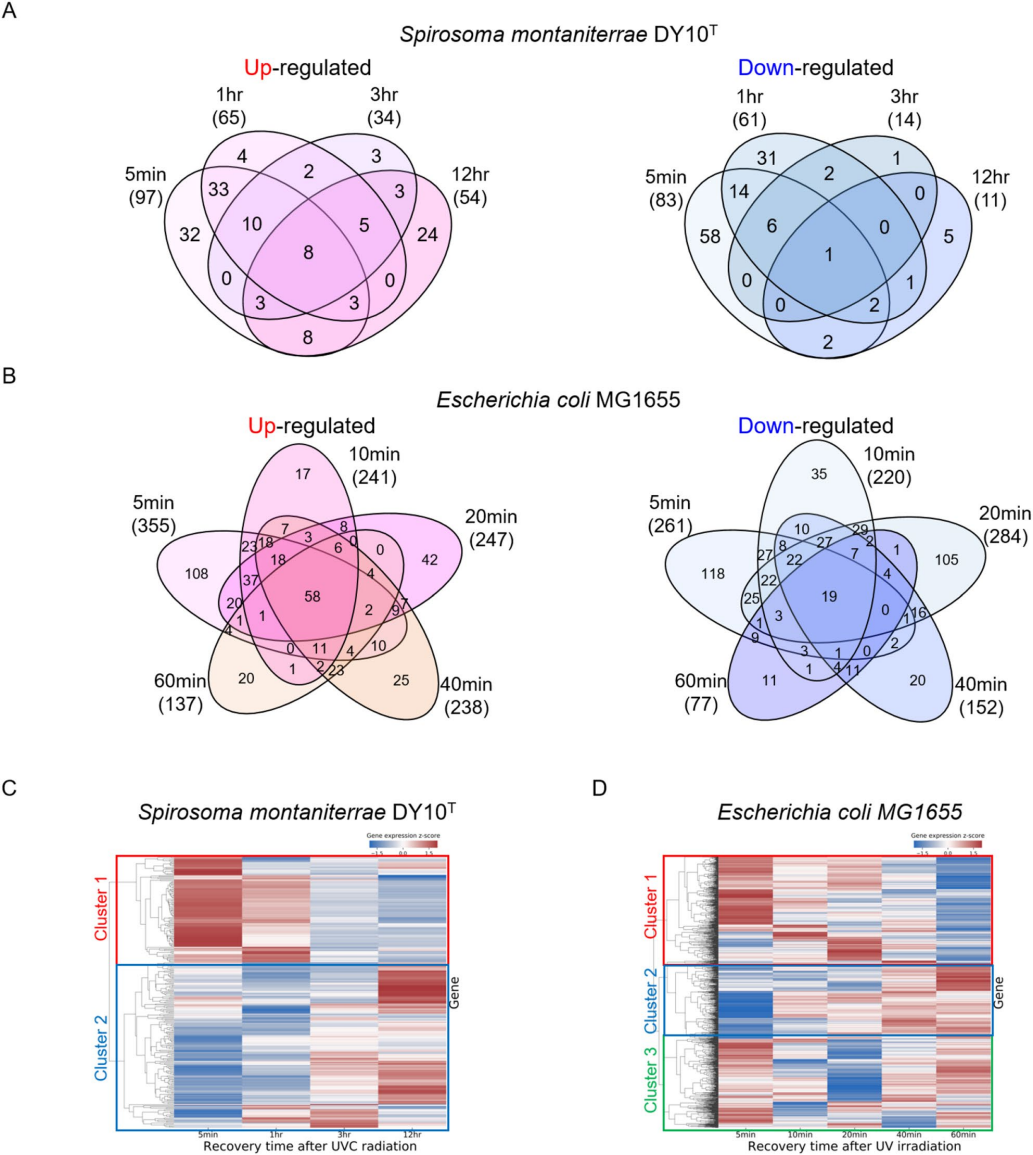
Differentially expressed genes (DEGs) analysis of *Spirosoma montaniterrae* DY10$^{T}$ and *Escherichia coli*. (**A**,**B**) The number of up-regulated and down-regulated DEGs with |$\log _{2} \text {(fold change)}$| $\ge$ 1 in varying time points. (**C**,**D**) Heatmap of 264 DEGs of *Spirosoma montaniterrae* and 1039 DEGs of *E. coli* in varying time points. The row represents the gene, and the columns represents each sample with varying time points. The redder the color, the higher the gene expression value. The bluer the color, the lower the gene expression value. Each bounding box identifies a gene cluster.

**Figure 3:**
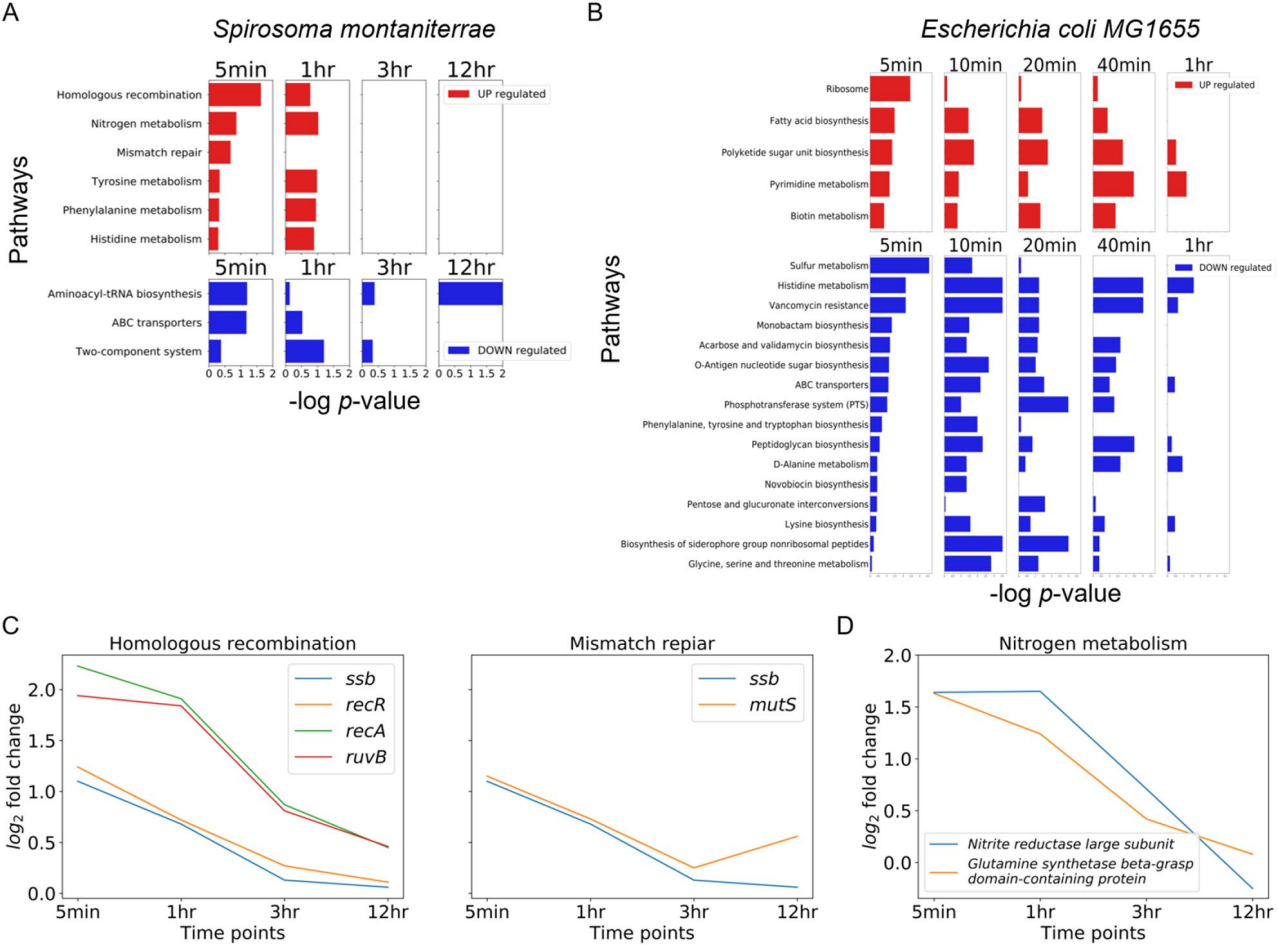
Pathway enrichment analysis of *Spirosoma montaniterrae* DY10$^{T}$ and *Escherichia coli*. (**A**) The significantly enriched terms of KEGG pathways of *Spirosoma montaniterrae* in varying time points. (**B**) The significantly enriched terms of KEGG pathways of *Escherichia coli* in varying time points. (**C**,**D**) Variation over time of significantly changed genes among genes related to homologous recombination, mismatch repair and Nitrogen metabolism processes in *Spirosoma montaniterrae*.

To investigate transcriptome-level responses to UVC radiation, we analyzed differentially expressed genes (DEGs) between the UVC untreated and UVC treated samples over time (|$\log _{2} \text {(fold change)}$| $\ge$ 1; Fig. 2A,B).Number of DEGs at 5 min, 1 h, 3 h, and 12 h after UVC radiation for *Spirosoma montaniterrae* was 97, 65, 34, and 54 for up-regulated and 83, 61, 14, and 11 for down-regulated, for a total of 264 unique genes. In the case of *E. coli*, the number of DEGs at 5 min, 10 min, 20 min, 40 min and 60 min after UVC radiation was 355, 241, 247, 238 and 137 for up-regulated and 261, 220, 284, 152 and 77 for down-regulated, respectively for a total of 1039 unique genes. The gene expression patterns of strain DY10$^T$ and *E. coli* were visualized through a hierarchically-clustered heatmap (Fig. 2C,D). We identified two clusters for strain DY10$^T$ and three clusters for *E. coli* with hierarchically-clustered heatmaps by Scipy package^26^ (Linkage threshold was set to 3). Cluster1 in strain DY10$^T$ contained 105 genes and was the set of genes that were up-regulated in early response, and cluster2 in strain DY10$^T$ contained 162 genes and was the set of genes that were down-regulated in early response (Fig. 2C). Cluster1 in *E. coli* contained 410 genes and was the set of genes that were up-regulated in early response, and cluster2 in *E. coli* contained 309 genes and was the set of genes that were down-regulated in early response (Fig. 2D). Cluster1 and cluster2 of each strain showed similar expression patterns. However, gene comparison based on sequence homology, 18 genes were shared in cluster1 and 33 genes were shared in cluster2. From these results, we confirmed that radiation-resistant strain and radiation-sensitive strain respond differently to UV radiation. Furthermore, we performed pathway enrichment analysis with KEGG pathway and DEGs to capture the biological implications of DEGs in strain DY10$^T$ and *E. coli*. The results showed that Homologous recombination, Mismatch repair (MMR) and Nitrogen metabolism were significantly enriched terms (*p*<0.05) among up-regulated genes in early response to UVC radiation in strain DY10$^{T}$ (Fig. 3A). In addition, we investigated the perturbations over time of the genes belonging to significantly enriched terms.

In Homologous recombination and Mismatch repair pathways, *ssb*, *recA*, *recR*, *ruvB* and *mutS* were remarkably up-regulated genes at an early response after UVC radiation and gradually decreased over time (Fig. 3C). *ssb* (AWR27_RS23025) is a gene that involved in both Homologous recombination and Mismatch repair process. Expression of *ssb* is a part of the SOS regulon and elevation of *ssb* gene in UVC radiated cells in an SOS-dependent manner was reported^27^. *recA* (AWR27_RS17265) and *recR* (AWR27_RS05955) play a central role in DNA maintenance and its induction is considered as a dominant marker for the onset of homologous recombination. When DNA damage occurs, bacterial RecR forms complexes with RecO and RecF that enhances loading RecA on to damaged DNA and catalyzes homologous recombination process^28–30^. We found it interesting that the strain DY10$^T$ also has RecR, RecF and RecO encoded genes but only *recR* was perturbed significantly. *ruvB* (AWR27_RS21175) is a gene encodes Holliday junction DNA helicase RuvB which is also key molecule for DNA maintenance especially in homologous recombination^31,32^. *mutS* (AWR27_RS05750) encodes a bacterial MMR protein that responsible for the repair of mispaired bases^33^. In Nitrogen metabolism, nitrite reductase large subunit (AWR27_RS22355) and glutamine synthetase beta-grasp domain-containing protein (AWR27_RS10835) are products of remarkably up-regulated genes at an early response after UVC radiation (Fig. 3D). Nitrite reductase (NiR) is an enzyme that catalyzes the reduction of nitrite (NO_2_$^{-}$) to nitric oxide (NO), thereby carrying out ionizing radiation-induced nitric oxide detoxification^34,35^. Glutamine synthetase (GS) is an ATP-dependent enzyme that catalyzes the assimilation of glutamate and ammonia to glutamine and the up-regulation of GS in bacteria is an effective means to survive when assaulted by ROS^36^.

Unlike strain DY10$^T$, among the up-regulated DEGs of *E. coli* in response to UVC radiation, the significantly enriched terms (Ribosome, Fatty acid biosynthesis, Polyketide sugar unit biosynthesis, Pyrimidine metabolism, and Biotin metabolism) were not related with DNA repair or ROS detoxification processes (Fig. 3B). This difference explains partially why *E. coli* is less resistant to radiation stress for survival.

### Regulatory mechanism in response to UVC radiation in *Spirosoma montaniterrae* DY10$^{T}$

**Figure 4:**
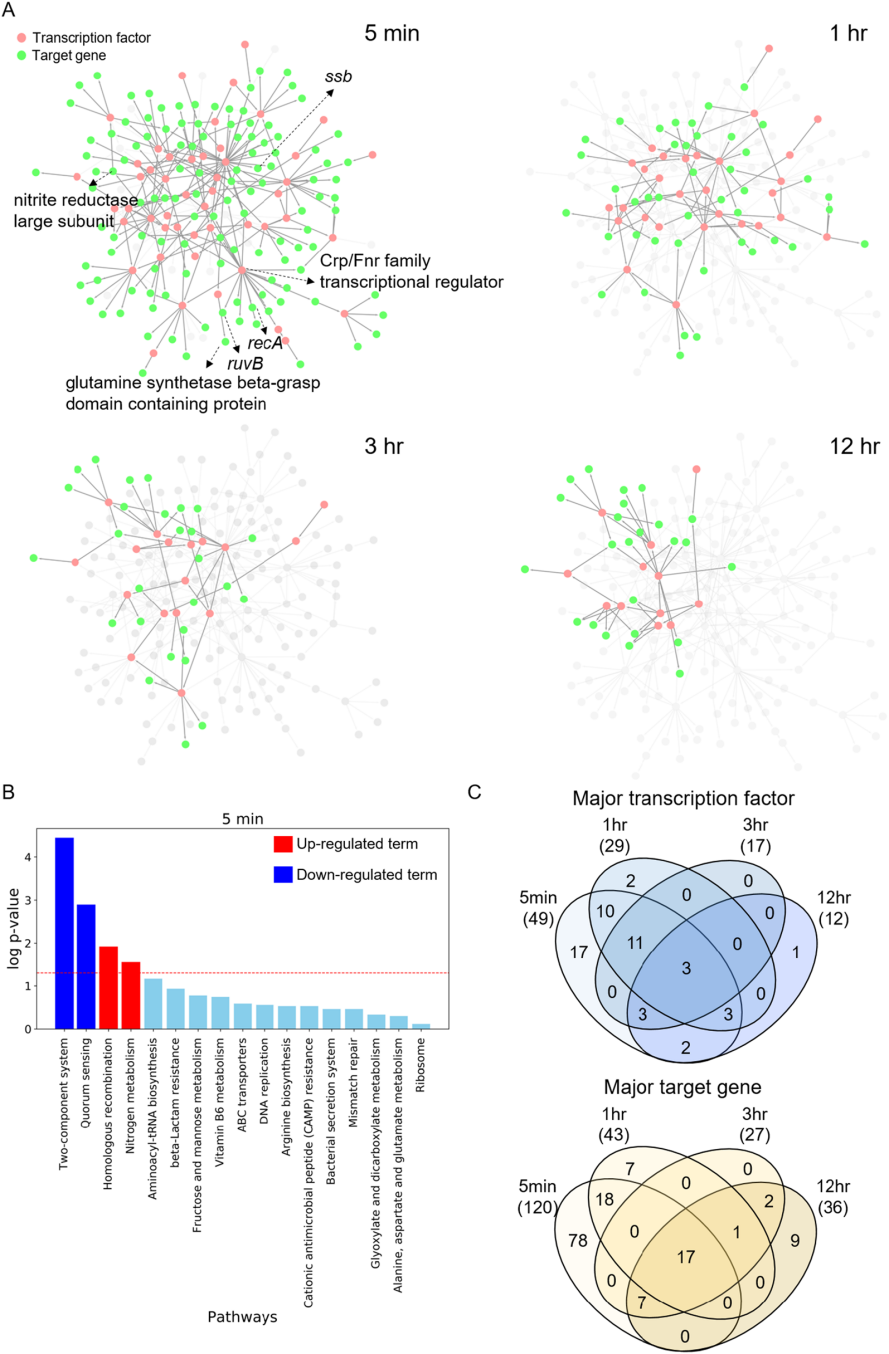
Transcriptional network module analysis. (**A**) Perturbation of transcriptional network module in varying time points. Major transcription factors are shown in red color and target genes are shown in green color. (**B**) Pathway enrichment analysis of genes in early response (5 min) module with KEGG pathways. Red colored bar represents up-regulated terms, blue colored bar represents down-regulated terms, and statistically insignificant terms are colored in light blue. (**C**) Venndiagrams show major transcription factors and major target genes over time identified by our analysis.

In order to understand the regulatory mechanism of strain DY10$^{T}$ in response to UVC radiation, we investigated time-varying transcriptional network module using PropaNet (“Materials and methods” section: “Gene regulatory network based analysis”) with GRN and time-series transcriptome data; untreated sample and UVC radiated samples collected at 5 min, 1 h, 3 h and 12 h after radiation. Transcriptional network module is a regulatory network of TFs and target genes that can describe state of organism at each time point. The results showed the visualization of identified time-varying transcriptional network modules in response to UVC radiation (Fig. 4A). The nodes and edges shown in the figure represent the condition-specific sub-network modules containing TFs and target genes that respond to UVC stimulation among the template GRN. Changes of the sub-network modules showed that the effects of UVC radiation gradually recovered over time. In order to further understand the biological implications of time-varying transcriptional network module, we performed pathway enrichment analysis with genes in each module and KEGG pathways. Homologous recombination, and Nitrogen metabolism was identified as significantly enriched and up-regulated terms at an early response (Fig. 4B). Homologous recombination involved genes in the early response module (5 min) were *recA* (AWR27_RS17265), *ruvB* (AWR27_RS21175), and *ssb* (AWR27_RS23025). Nitrogen metabolism involved genes in the early response module (5 min) were nitrite reductase large subunit (AWR27_RS22355), and glutamine synthetase beta-grasp domain containing protein (AWR27_RS10835). Additionally, Two-component system and Quorum sensing pathway were identified as significantly enriched and down-regulated terms at the early response module. Biological implications for all time points were shown in Supplementary Fig. S2. When analyzing the regulator of each network module, 49 TFs were identified as major TFs in early response. Over time, different target genes in the gene regulatory network were regulated by different sets of TFs, leading the cell to different states (Fig. 4C).

**Figure 5:**
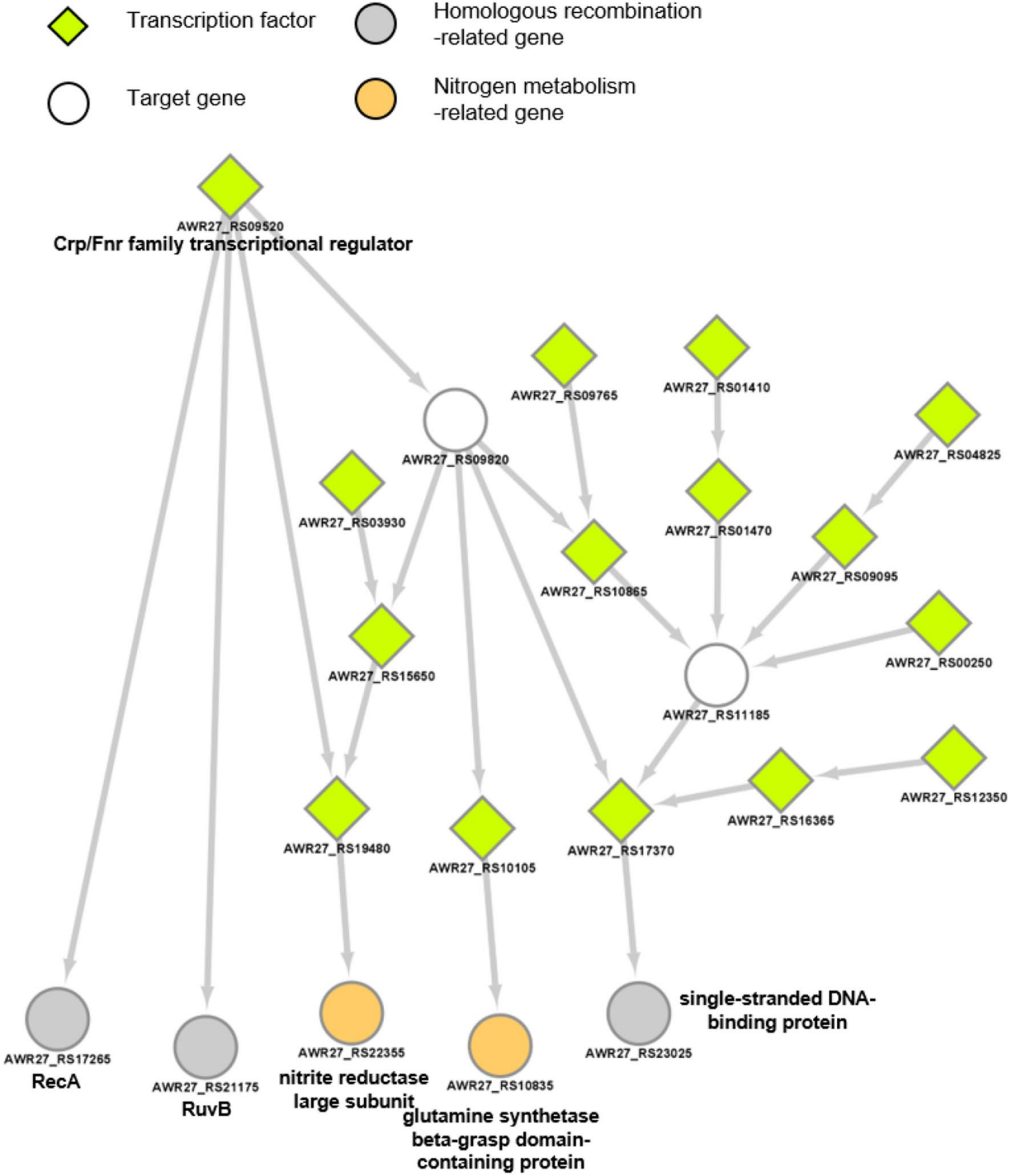
Homologous recombination and Nitrogen metabolism-related transcriptional network module in early response of *Spirosoma montaniterrae* DY10$^T$ to UVC radiation. Network represents regulatory relation between genes. In the network, diamond shaped nodes are transcription factors and circled nodes are target genes. Grey colored nodes are Homologous recombination-related genes and orange colored genes are Nitrogen metabolism-related genes.

The key to regulatory network based analysis is regulatory mechanism with identified major transcription factors and their target genes by time. In the DEGs analysis, we identified 180 genes that were significantly affected by UVC radiation. Through the regulatory network-based analysis, we prioritized 120 target genes in early response to UVC stimulation, and additionally identified regulatory relationships with 49 regulatory factors. In particular, significantly up-regulated pathways (Homologous recombination and Nitrogen metabolism) in the 5-min module were analyzed in detail and visualized (Fig. 5). The result showed the Crp/Fnr family transcriptional regulator (AWR27_RS09520) is a master regulatory TF that initiates expression changes of *recA*, *ruvB*, *ssb*, nitrite reductase large subunit, and glutamine synthetase beta-grasp domain containing protein directly or indirectly. Crp/Fnr family transcription regulators typically function as transcriptional activators in several bacterial species and regulate fumarate reductase, nitrate, and nitrite reductase to respond to intracellular and extracellular signals such as temperature, oxidative and nitrative stress, and nitric oxide^37^.

As a result, both the DEGs based analysis and the regulatory network based analysis identified that Homologous recombination and Nitrogen metabolism pathways were the most significant processes for radiation-resistance in strain DY10$^T$. However, the DEG based analysis showed only quantitatively meaningful genes. Meanwhile, the regulatory network based approach identified the master regulatory TF of radiation-resistance mechanism. In addition, our approach identified the other 17 radiation-resistance mechanism related genes and their regulatory interactions that could not be captured by the gene-level quantitative approach.

### Operons involved in early response to UVC radiation

Operon is an important genetic regulatory system found in bacteria. The genes in operon are co-transcribed under the control of a single promoter and are often functionally related^38^. By analyzing the operon, we can understand how the expression of genes in organism is efficiently regulated in response to external stimuli. It is an important part of understanding the complex regulatory system of bacteria. operonSEQer^39^ is a statistics and machine learning based algorithm that predicts relevant operon pair with signals from RNA-seq data across two genes. Unlike other existing tools, operonSEQer is a flexible tool that does not use functional relationship information and showed remarkable operon predictive performance to bacterial strains that have not been studied.

**Figure 6:**
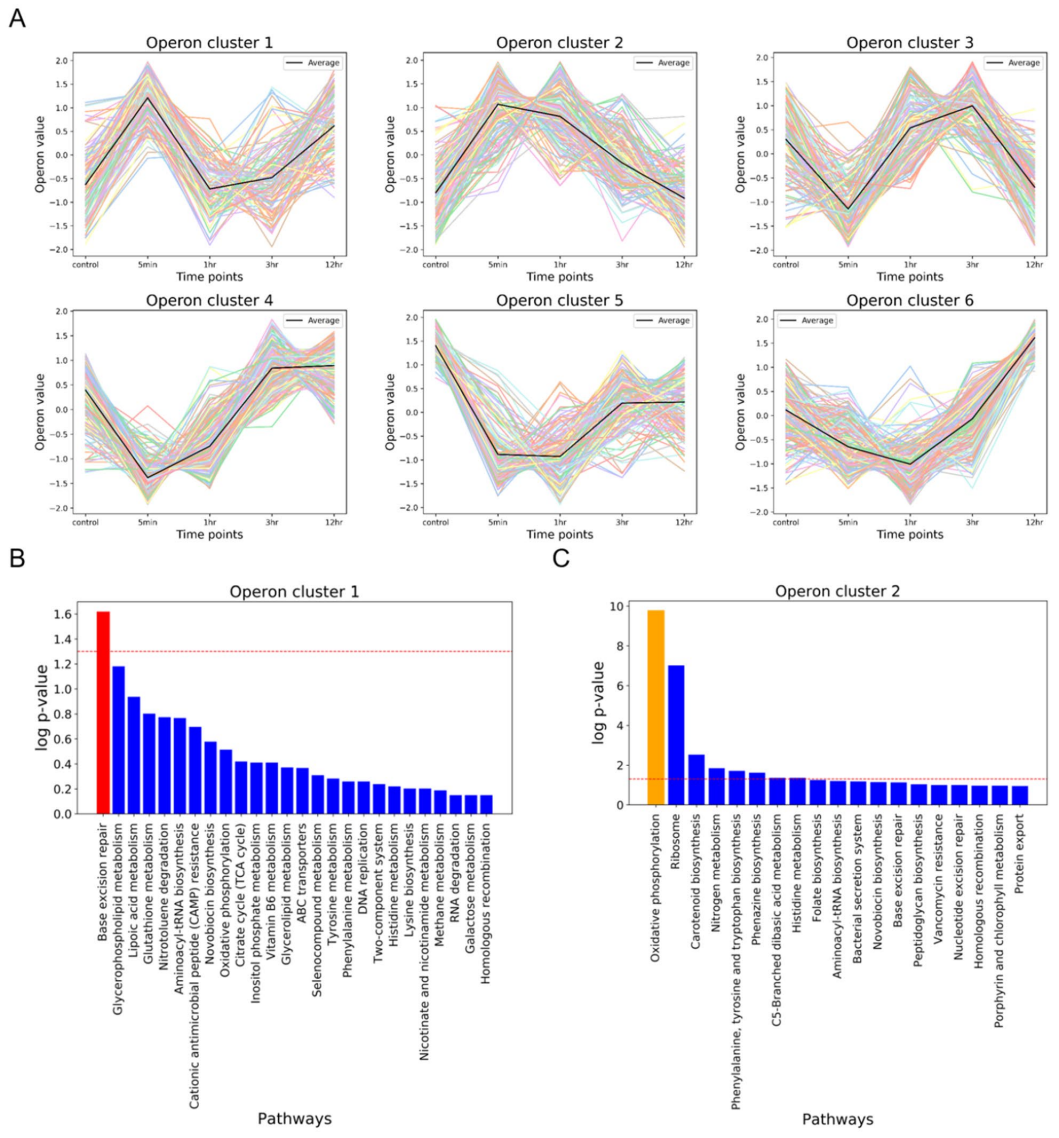
Operon prediction and analysis. (**A**) Six operon clusters were identified with k-means clustering method. Black colored line is the average of the operon values that belonging to the cluster at the each time points. (**B**,**C**) Pathway enrichment analysis of genes in operon cluster 1 and 2. Red colored bar is most enriched term in cluster1 and orange colored bar is most enriched term in cluster2. The red dotted line indicates statistical significance of *p*-value.

In this study, we identified 1113 operons in *Spirosoma montaniterrae* and classified them into six clusters with similar operon value perturbation patterns (Fig. 6A). To understand the function of operons involved in initial response to UVC radiation, we conducted pathway enrichment analysis with genes in operon cluster1 and operon cluster2 that initially up-regulated and gradually decline over time. The results showed that the most significant term of genes in operon cluster1 is a Base excision repair (Fig. 6B) and the most significant term of genes in operon cluster2 is an Oxidative phosphorylation and nitrogen metabolism was also included in significant terms (Fig. 6C). Base excision repair is also involved in DNA repair process induced by UVC radiation like Homologous recombination and Oxidative phosphorylation is also biological process that involved in ROS scavenging^40–42^. Among the operons involved in the initial reaction, three operons contain Base excision repair-related genes and eight operons contain Oxidative phosphorylation-related genes (Fig. S1). We combined GRN based regulatory information with predicted operons, two of the eight Oxidative phosphorylation-related operons have the binding site of the master regulatory TF identified in the previous analysis in front of the operon. Thereby those two operons have potential to be an another target regulatory gene set of master regulatory TF in early response. As a result, some of DNA repair and ROS scavenging processes-related genes in strain DY10$^T$ are located close to each other on the genome and are regulated efficiently by forming operons.

## Discussion

In radiation-resistance species, such as *Deinococcus* sp., DNA repair and ROS scavenging processes have been experimentally demonstrated as major mechanisms for survival under UVC radiation. However, due to the genetic diversity between species or genera^43^ of bacteria, biological mechanisms of DNA repair and ROS scavenging processes can be significantly differ in diverse bacteria species. Thus, straightforward DEG analysis may not be sufficient for identifying DNA repair and ROS scavenging processes in uncharacterized genomes such as strain DY10$^T$ in our study. Furthermore, little is known about the regulatory mechanisms for survival under UVC radiation. Therefore, we sought to understand the radiation-resistance characteristic in terms of biological regulatory system by constructing homology based GRN and identifying operons with six computational biology techniques.

Gene expression level comparison identified that 180 genes (97 up-regulated and 83 down-regulated) are involved in the early response of strain DY10$^T$ to UVC radiation and that the gene set changes over time. With gene-level analysis, we identified condition-specific expression perturbed gene sets. However, gene-level approach cannot capture global patterns of gene expression that are important for understanding complex biological processes. To complement this limitation, pathway-level analysis was conducted. Pathway-level approaches capture a more comprehensive view of biological processes by integrating information from multiple genetic and molecular pathways. We observed that Homologous recombination, Mismatch repair and Nitrogen metabolism were significantly altered processes in the early response of strain DY10$^T$ to UVC radiation. Homologous recombination is an important DNA repair process that repairs damaged DNA sequences using identical or very similar undamaged DNA sequences as templates. Recombinational DNA repair is both the most complex and the least understood of bacterial DNA repair processes^44^. MMR also contributes to genome stability by mediating DNA damage signaling in response to DNA damage^45^. MMR proteins have a role in the imposition of cell cycle check-points and apoptotic signaling in response to UVC radiation. One mechanism of the MMR-induced DNA damage response is that MMR proteins recognize sites of damage and recruit components of the DNA damage response pathway either directly or *via* interactions with other entities^46^. Nitrogen metabolism is a basic biological process of nitrogen cycle and plays a crucial role in removing ROS in the cell. When cells are exposed to UVC radiation, nitric oxide and ROS are formed and react with hydroxyl radical-induced adenine radicals. This reaction forms common DNA damage bases (hypoxanthine and 8-azaadenine) and induces DNA double-strand breaks^47^. Our results showed that strain DY10$^T$ repair damaged DNA and remove ROS together to increase viability in early response to UVC radiation.

Gene-level and pathway-level analysis are highly annotation-biased approach, whereas network-based approaches focus on associations between genes rather than relying on annotations. By constructing time-varying transcriptional network modules, we identified which genes were regulated and how those genes were regulated during UVC damage recovery. In particular, we identified DNA repair and ROS scavenging processes are key mechanisms for UVC induced damage recovery in strain DY10$^{T}$ as in other radiation-resistant bacterium. Furthermore, we first revealed that Crp/Fnr family transcription regulator (AWR27_RS09520) is a master regulator TF that regulates the initial activation of UVC induced damage recovery processes of strain DY10$^T$. The function of Crp/Fnr family transcription regulator as a regulator of nitrite reductase with ROS scavenging function has also been found in other species^37^. However, its role as a regulator of the DNA repair system has not been well studied. In addition, two genes (AWR27_RS09820 and AWR27_RS11185) in the regulatory module did not show significant expression changes in response to UVC radiation and their functions are unknown. However, they were captured as intermediate genes in regulatory module and it is possible that it has functions involved in DNA repair and ROS scavenging processes through regulatory network based analysis. Operon is a major regulatory system in bacteria^48^, and operon analysis also showed the genetic response to UVC in terms of systems. Our results showed that genes involved in DNA repair and ROS scavenging processes are located close to each other on the genome and are efficiently regulated. Similar to network-based approach, operon analysis provides intuition about the function of genes belonging to an operon but whose function is unknown.

## Conclusion

In this study, we characterized the underlying mechanisms for radiation-resistance by constructing gene regulatory network of an uncharacterized bacterium strain DY10$^T$. In particular, we identified major regulatory TFs for radiation-resistance in a computational framework. It is interesting that a large number of genes regulated by the TFs identified are not well characterized, thus we believe that identifying functions of these genes can be valuable new knowledge for the mechanisms for UVC resistance. Consequently, our biological system-level approach can help bridge the gap between the transcriptome-level and the organism-level and enable interpretsation of complex biological processes under diverse conditions.

On the other hand, there are two limitations in our approach. First, PropaNet depends on reliable TF-target interactions. GRN of the strain DY10$^T$, is inferred based on homology with genomic sequence of bacteria in UniProt database and it has potential to contain false positive TF-target interactions. To overcome this limitation, validation through additional complicate experiments is required. The most widely used method for identifying TF-target interaction is TF ChIP-seq. With TF ChIP-seq, binding sites and DNA sequence motif for TFs can be identified precisely. Second, our framework construct GRN that do not consider operon system in bacteria. Operon is a very important gene regulatory system in bacterial gene network. In previous studies, it was found that more than 50% of all genes in *E. coli* are organized into operons^49^. In this study, 66% of all genes in strain DY10$^T$ were predicted to be regulated by the operon system. To capture more meaningful underlying mechanisms in bacteria, developing operon system considered bacterial GRN inference method can be future work.

## Materials and methods

### Cell growth, radiation and RNA extraction

The bacterial strain DY10$^{T}$ (KCTC 23999$^{T}$) was grown at $25\,^{\circ }\textrm{C}$ in liquid nutrient-rich medium tryptone glucose yeast extract (TGY; 1% tryptone, 0.1% glucose and 0.5% yeast extract) or TGY agar plates^23^. For the UVC treatment, the early stationary phase cells on TGY agar plates were exposed to 600 JM$^{-2}$ of ultraviolet using an ultraviolet crosslinker (UVP, CX-2000, CA, USA) at 254 nm^50,51^. The post-radiation cells of UVC were collected by centrifugation (17,500×*g* for 5 min) at 5 min, 1 h, 3 h and 12 h with washing and stored with RNAlater solution at $-80\,^{\circ }\textrm{C}$. Total RNA was isolated using the Ambion Ribopure-Yeast RNA kit, according to manufacturer’s instructions.

### Library preparation and gene expression analysis

Strand-specific RNA-seq library preparation and sequencing was carried out as a paid service by Somagenics Inc., Santa Cruz, California, USA. Paired-end reads (Illumina NextSeq 500 v2, 2 $\times$ 150 bp, 3 Gb average output per sample) were obtained from strain DY10$^{T}$. Each sample was obtained from three independent RNA isolations, for each strain. Reads of each samples’ adaptor trimmed by Trim galore (version 0.6.7)^52^ for quality control. Clean reads of the samples were aligned against the strain DY10$^{T}$ reference genome (Build: ASM198895v1) using Hisat2 (version 2.1.0)^53^. HTSeq (version 0.12.4)^54^ was used to quantify the mapped reads per ORF. Differentially expressed genes were identified with a $\log _{2} \text {(fold change)}$ threshold of − 1 and 1 (|$\log _{2} \text {(fold change)}$| $\ge$ 1). For radiation-sensitive (negative) strain, UVC radiated *E. coli* with multiple time points gene expression data (GSE9) downloaded from GEO (Gene Expression Omnibus).

### Homology based reconstruction of Gene Regulatory Network (GRN)

To determine the regulatory interactions between genes in strain DY10$^T$ with genome and protein sequence information, We used four computational methods. GRN construction operates in five steps as below. In the entire process, homology to known prokaryotic sequences is an important concept to reflect the regulatory mechanisms present in prokaryotic organisms and has been used for TF screening and TF binding site prediction^55^.

- *Step 1. Transcription factor prediction.* DeepTFactor^56^ is a state-of-the-art deep learning based tool that learns DNA-binding domains of TFs in several bacteria species and predicts whether a query protein is a TF. In this study, pre-trained DeepTFactor^56^ was used for identifying potential TFs using protein sequences from strain DY10$^{T}$. Protein sequences from strain DY10$^{T}$ were used as input queries of model and model returns whether a query protein is a TF.
- *Step 2. Homology based transcription factor screening.* BLAST^57^ is a widely used local alignment tool. Using the program blastp with the default parameters, among the potential TFs predicted by DeepTFactor, potential TFs that are statistically similar (e-value < 0.05) to known prokaryotic TFs included in the prokaryotic TF database were selected. Sequences of known prokaryotic TFs were collected from UniProt^58^ database based on strains in PRODORIC^59^ database.
- *Step 3. Prediction of accessible genome regions for each transcription factors.* HOMER^60^ is a motif discovery algorithm that searches accessible genome regions for TFs using the position weight matrix (PWM) of TFs. We computed the genome binding sites of screened TFs of strain DY10$^T$ using HOMER. PWM of each TF of strain DY10$^T$ is derived from PWM of the most similar TF among the TFs present in the PRODORIC database.
- *Step 4. Identification of target gene of transcription factors.* Bedtools^61^ is a genomic analysis tool. We identified the target genes of the selected TFs by analyzing the computed accessible genomic regions and its down-stream genes with bedtools. Through the bedtools closest command of bedtools v2.30.0, the gene closest to the binding site of each TF was identified and assigned as the target gene of TF.
- *Step 5. Reconstruction of Gene Regulatory Network* With identified TFs and their target genes, we reconstructed a GRN of strain DY10$^{T}$. GRN is a bipartite graph where source nodes are TFs and target nodes are target genes of TFs.

### Gene regulatory network based analysis

Ahn et al. developed a PropaNet^13^, a state-of-the-art time-series trancriptome analysis method. PropaNet calculates the impact of each TF on the target genes at each time point using influence maximization and network propagation^62,63^ techniques with template GRN. The calculated impact of each TF is used to select the major TFs for each time points by prioritizing them. A regulatory network was constructed for each time point using the selected major TFs and their target genes. The constructed time-varying regulatory networks allow interpretation of the regulation of key mechanisms for UVC radiation response. In this study, a constructed GRN of strain DY10$^T$, time-series expression profile, DEGs list in each time point and TF list of strain DY10$^T$ were used as inputs of PropaNet and time-varying networks were obtained and additional analysis were conducted.

### Operon prediction and clustering

Krishnakumar et al. developed a operonSEQer^39^, a state-of-the-art statistic and machine learning based algorithm that predicts bacterial genome operon. operonSEQer shows remarkable predictive power using only RNA-seq data and genomic features. In this study, we identified operons in *Spirosoma montaniterrae* using operonSEQer with follow criteria. Among the six machine-learning models included in operonSEQer, 3 or more models predicted gene pairs as operon were selected as being in the same operons.

Predicted operons were classified into six clusters according to the pattern of operon value change over time. *i*-th operon ($O_{i}$) is represented as a set of operon values listed in time order and the operon value at time *t* ($O_{i,t}$) is the average of z-score values of *n* genes({$g_{i,t,1}$, $\cdot$ $\cdot$ $\cdot$, $g_{i,t,n}$}) at time *t* belonging to the *i*-th operon. For clustering, we used the scikit-learn module of python3 to implement the K-means clustering algorithm^64^. In this study, we divided a set of operons ($O_{i}$) into six disjoint clusters.

$$O_{i} = [O_{i,control}, O_{i,5\,min}, O_{i,1\,h}, O_{i,3\,h}, O_{i,12\,h}]$$

$$G_{i,t} = \{g_{i,t,1}, ..., g_{i,t,n}\}$$

$$O_{i,t} = \frac{1}{n}\sum _{j=1}^{n}{g_{i,t,j}}, \quad g_{i,t,j} \in G_{i,t}$$

where $O_{i}$ is a set of operon values listed in time order of *i*-th operon, $G_{i,t}$ is a set of expression level of genes included in *i*-th operon at time *t*, $g_{i,t,j}$ is a expression level (z-score) of gene included in $G_{i,t}$.

### Pathway database

For pathway enrichment analysis, we used biological pathway information obtained from the Kyoto Encyclopedia of Genes and Genomes (KEGG) database^65^. We used 117 pathways and 1051 genes for *Spirosoma montaniterrae* DY10$^{T}$ and 121 pathways and 1705 genes for *E. coli* from the KEGG database.

## Supplementary Information

Supplementary Information.

## Data Availability

The datasets generated and analysed during the current study are deposited in the Gene Expression Omnibus (GEO) repository. Data accession link (GSE223604): https://www.ncbi.nlm.nih.gov/geo/query/acc.cgi?acc=GSE223604.
