# Supplementary material for: Characterization of radiation-resistance mechanism in Spirosoma montaniterrae DY10T in terms of transcriptional regulatory system
Source: Sci Rep. 2023 Mar 23;13:4739. doi: 10.1038/s41598-023-31509-8 (PMC10036542; doi:10.1038/s41598-023-31509-8)
Supplement: Supplementary file 1 — Supplementary Information. [file 41598_2023_31509_MOESM1_ESM.pdf]

Process	Tool	Input	Output	Description
Gene Regulatory Network reconstruction	DeepTFactor	Protein sequences	Predicted TFs	Prediction of TFs in the organism
	BLAST	Predicted TFs & Known prokaryote TFs	Prokaryote homologous TFs	Screening prokaryotic TFs from predicted TFs
	HOMER	Position Weight Matrix & Reference genome	Genomic accessible regions	Binding site search for each TFs
	Bedtools (closest)	Genomic positions	Nearest genes	Assignment of target genes for each TFs
Network module analysis	PropaNet	Time-series transcriptome profiles & GRN	Major TFs & target genes in varying time points	Construction of time-varying regulatory networks in response to UVC
Operon analysis	operonSEQer	Read coverage	Gene pairs in the same operon	Prediction of the operons of genome

Table S1. Summary of the dataflow for gene regulatory network inference and system-level analysis.

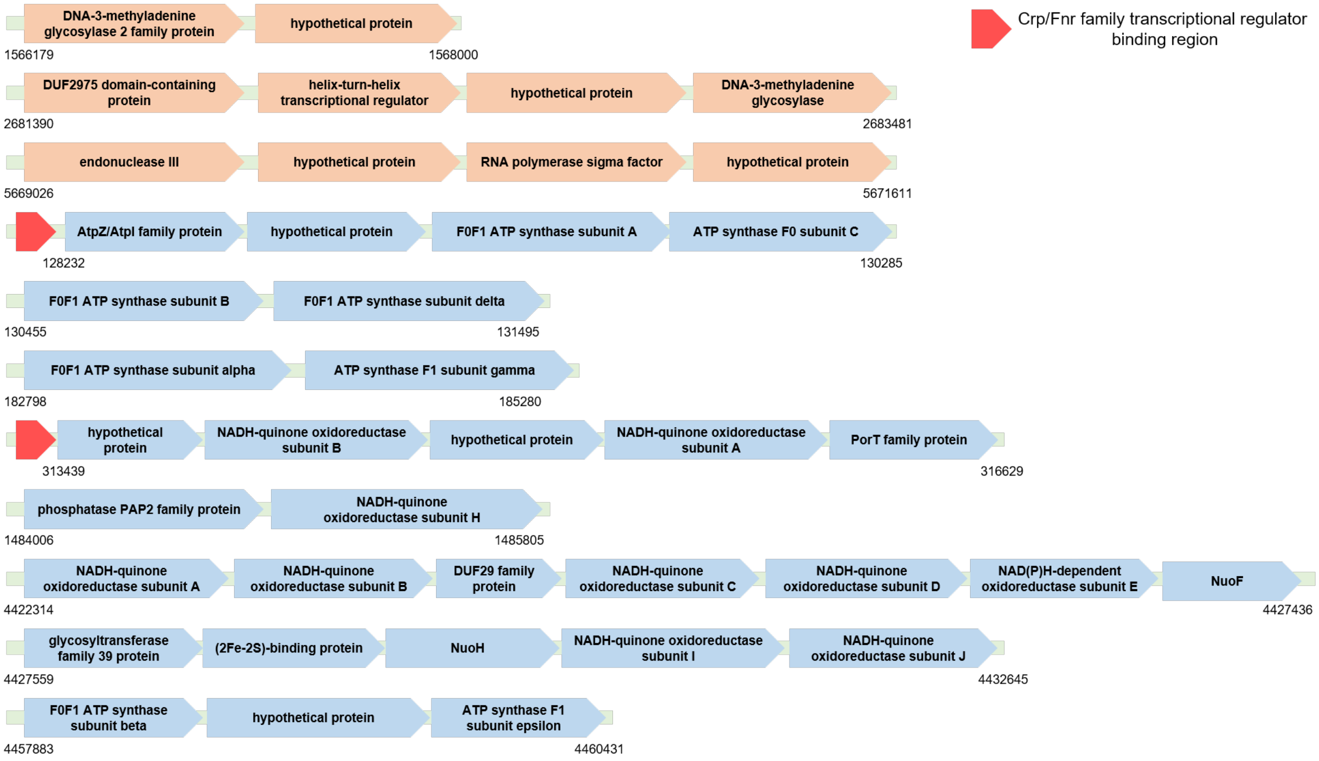

Figure S1. Visualization of operons involved in the early response and including DNA repair and oxidative phosphorylation-related genes. Top three operons contains Base excision repair-related genes. The other eight operons contains Oxidative phosphorylation-related genes. The numbers below each operon indicate the start and end positions of the operon. Red colored marker indicates the binding region of Crp/Fnr family transcriptional regulator.

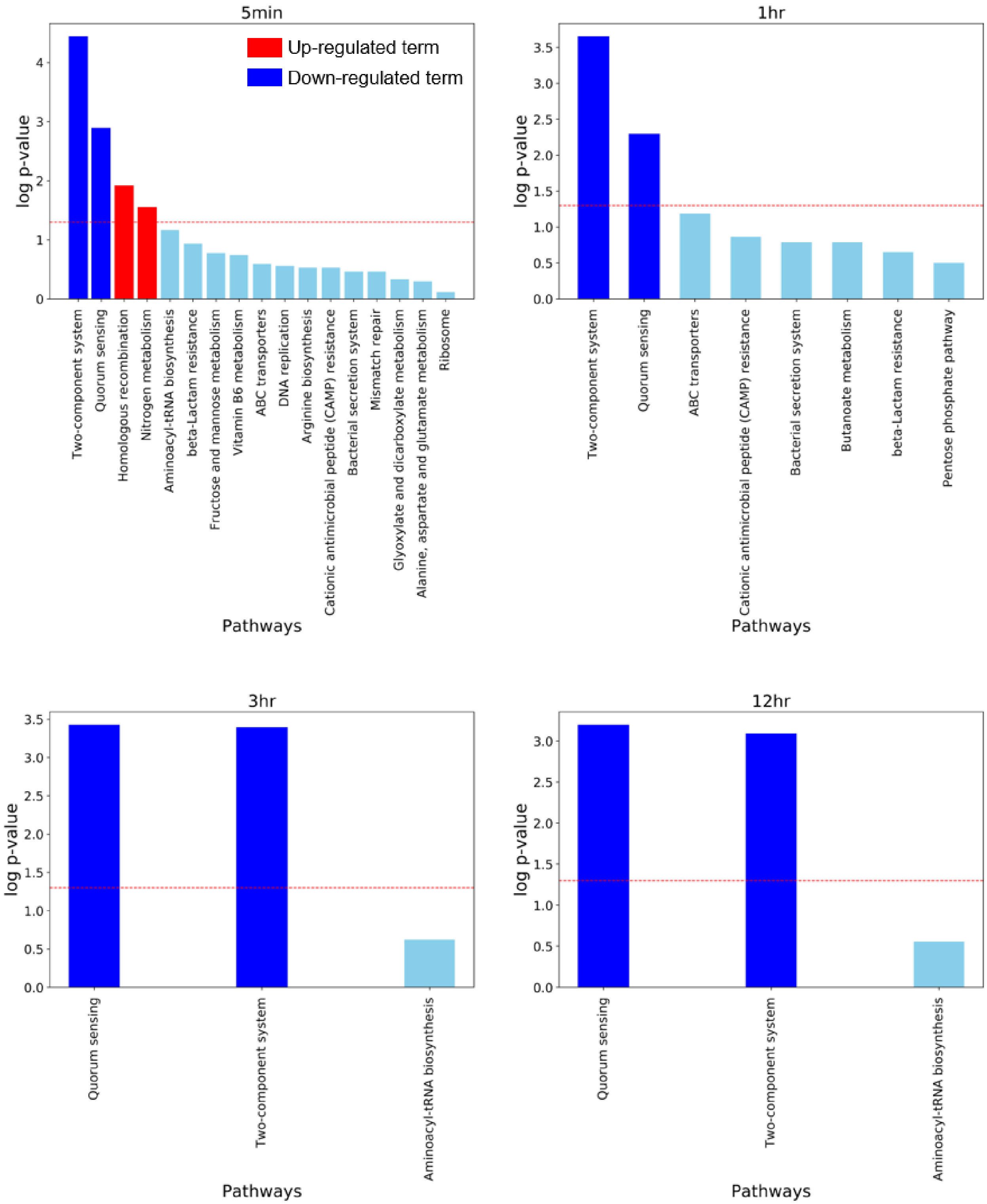

Figure S2. Visualization of pathway enrichment analysis with time-varying transcriptional network modules. Pathway enrichment analysis of genes in early response (5min) module. Red colored bar represents up-regulated terms, blue colored bar represents down-regulated terms, and statistically insignificant terms are colored in light blue.
